# The rediscovery of *Rhodnius domesticus* Neiva & Pinto, 1923 (Hemiptera: Reduviidae: Triatominae) in the state of Espírito Santo, Brazil

**DOI:** 10.1590/0037-8682-0323-2020

**Published:** 2020-12-21

**Authors:** Guilherme Sanches Corrêa-do-Nascimento, Danielle de Oliveira Moreira, Cleber Galvão, Claudiney Biral dos Santos, Aloísio Falqueto, Gustavo Rocha Leite

**Affiliations:** 1 Universidade Federal do Espírito Santo, Unidade de Medicina Tropical, Maruípe, Vitória, ES, Brasil.; 2 Instituto Nacional da Mata Atlântica, Santa Teresa, ES, Brasil.; 3 Fundação Oswaldo Cruz, Instituto Oswaldo Cruz, Laboratório Nacional e Internacional de Referência em Taxonomia de Triatomíneos, Rio de Janeiro, RJ, Brasil.

**Keywords:** Insect vectors, Rhodnius, Triatominae, Chagas disease, Animal distribution, Brazil

## Abstract

**INTRODUCTION:**

*Rhodnius domesticus* Neiva & Pinto, 1923 is a rare sylvatic triatomine endemic to the Atlantic Forest, with one known record for Espírito Santo (ES), Brazil from 1969. We present here its rediscovery in ES, 42 years after its first record.

**METHODS:**

In January 2011, a triatomine specimen was collected from a rural area of the municipality of Santa Teresa, ES.

**RESULTS:**

We confirmed this as a new record of *R. domesticus* in the Baixo Caldeirão locality.

**CONCLUSIONS:**

This finding supports the possibility of a wild population of *R. domesticus* in the mountainous region of the Atlantic forest of ES.

Chagas disease, an infection caused by the protozoan parasite *Trypanosoma cruzi* Chagas, 1909*,* was originally a sylvatic enzootic infection that began to be a risk when some triatomine vectors became domiciliated^1^. However, predominant sylvatic and less-studied triatomines can invade and sporadically colonize anthropic habitats^2^. As these invasions theoretically start the process of domiciliation, studies on sylvatic triatomine species are important for controlling Chagas disease^3^ ^,^ ^4^.

Species of the subfamily Triatominae are blood-sucking insects that are vectors of Chagas disease, which is transmitted to humans and other mammals through the feces and urine of infected triatomines, usually immediately after a blood meal. Currently, this group of vectors consists of 151 extant and three fossil species assigned to five tribes^5^. The tribe Rhodniini contains two genera, *Rhodnius* Stål, 1859 and *Psammolestes* Bergroth, 1911^6^. Despite their different morphologies and ecological habits, both are mainly arboricolous. The genus *Rhodnius* is well-characterized and can be easily differentiated from other triatomine genera due to the presence of apically inserted antennae and distinct callosities behind the eyes^7^. On the other hand, their species are almost identical^8^.

*Rhodnius domesticus* is a species that, despite what is suggested by its specific epithet, essentially has sylvatic habits and it occasionally invades human domiciles possibly when attracted by artificial light sources. Researchers have proposed that the species is associated with bromeliads, palms, and marsupial and rodent nests^9^. However, several of the very few reported records for the species are from domestic and peridomestic areas^10^. 

*R. domesticus*, endemic to the Brazilian Atlantic forest, has already been reported in the Brazilian states of Bahia, ES, Minas Gerais, Paraná, Rio de Janeiro, São Paulo, and Santa Catarina^6^. Further knowledge about the natural habits of *R. domesticus* is required to understand species distribution. *Rhodnius zeledoni* Jurberg, Rocha & Galvão, 2009, which seems to be identical to *R. domesticus*, was described based on only one very damaged specimen found in the state of Sergipe, Brazil, a region possibly included within the distribution of *R. domesticus*. Therefore, the examination of further material is essential to confirm whether *R. zeledoni* is a valid species^6^.

To date, ES has a single known occurrence record of *R. domesticus* from 1969 in the municipality of Alfredo Chaves^11^. Since that first and unique record, the species was not found again in the state. We present here the rediscovery of *R. domesticus* in the state of ES, 42 years after its first and single record.

In January 2011, a local person captured a triatomine specimen in a chicken coop next to his residence in a rural area of the locality of Baixo Caldeirão, municipality of Santa Teresa, ES (19.9138°S and 40.7517°W) (Figure 1). We investigated the site of the record and its surrounding areas, but we could not find another specimen. Since there was no evidence of colonies in the domestic and peridomestic areas, it seemed likely that the insect was dispersed from adjacent forests.

**FIGURE 1:**
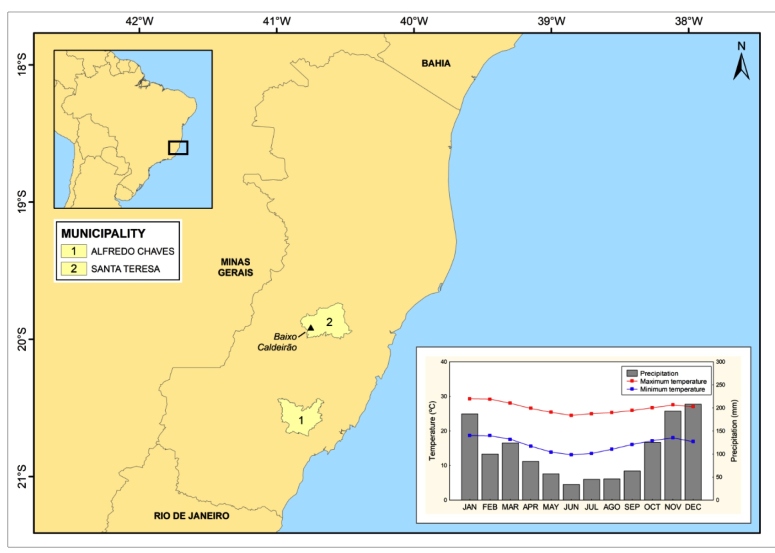
Map of the state of Espírito Santo, Brazil, showing [1] the municipality of Alfredo Chaves, where *R. domesticus* was found for the first time in 1969, and [2] the locality of Baixo Caldeirão, municipality of Santa Teresa, where we report its rediscovery 42 years after its unique register in ES. The monthly maximum and minimum temperatures and precipitation for the locality are presented in the map graph inside^12^.

We identified the collected specimen as a female of *R. domesticus* in the Laboratory of Entomology of the Espírito Santo State Department of Health. Specialists of the National and International Laboratory of Taxonomy of Triatominae of the Oswaldo Cruz Institute, Fiocruz (Rio de Janeiro, Brazil) confirmed the taxonomic identity of the species. Because the insect was dead and dry, we could not test for *T. cruzi* infection.

The collected specimen presented a length of 15 mm, a maximum width of the pronotum of 3.64 mm, and a maximum width of the abdomen of 5.92 mm. The length of the head was approximately 2.5 times the width across the eyes (1:0.43), and it was slightly longer than the pronotum (1:0.96). The anteocular region was three times longer than the postocular region (1:0.33). The ratio of the width of the eye to the synthlipsis was 1:1.43 (Figure 2). The specimens presented compatible ratios but with slightly smaller measurements than those provided by Lent and Wygodzinsky^7^ and Neiva and Pinto^8^ in the original species description; in a general way, the features were consistent with those provided by them. A short *R. domesticus* female, 15.5-mm-long, was also recently collected in Minas Gerais^10^.

**FIGURE 2:**
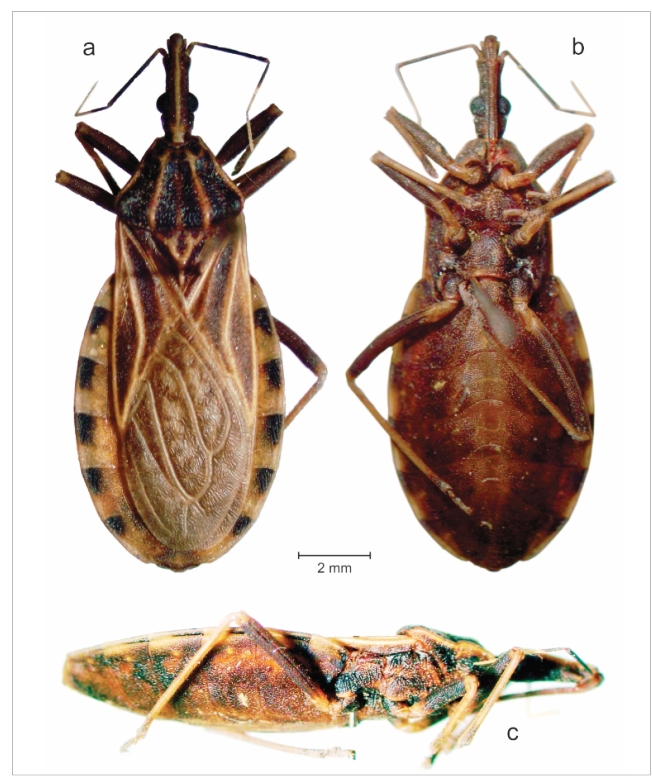
Original photographs of the *Rhodnius domesticus* female specimen collected in the locality of Baixo Caldeirão (19.9138°S and 40.7517°W), Santa Teresa, Espírito Santo, Brazil, in January 2011, showing it in (a) dorsal, (b) ventral, and (c) lateral view.

The area where the specimen was collected, the mountainous region of ES, is occupied by small familiar crops and forest fragments. It has an irregular terrain with an approximate elevation of 500 m above sea level, characterized by mild temperatures with an average annual temperature of 21 °C. The locality is in a transitional zone between tropical rainy and dry climates, with an approximate annual rainfall of 1,200 mm^12^. The site was characterized as a rocky area (Figure 1).

Surprisingly, there was a gap of 42 years between the two known *R. domesticus* records in ES. This made us reckon that either the 1969 record was an error, or that the species no longer existed in that region. For example, during 10 years of fieldwork, between 1996 and 2005, collectors captured almost 4,000 triatomines in ES, and none of them was *R. domesticus*. Several captures occurred in Alfredo Chaves and Santa Teresa, the only two municipalities in ES where *R. domesticus* is now known^13^. This may indicate that *R. domesticus* is a specialist species with small and restricted populations because of its biological and ecological characteristics, such as its occurrence in habitats formed by epiphytic bromeliads in association with specific small mammal nests^14^. In addition, artificial light may not attract it with the same intensity at which it attracts other triatomine species^13^.

Both the known occurrences of *R. domesticus*, even though collected over 40 years apart, are located in relatively close areas, suggesting a wild population in ES. The confirmation of the species occurrence is important for two main reasons: ES may comprise areas with one of the highest *T. cruzi* infection rates of triatomines ever recorded^15^, and *R. domesticus*, distributed largely in the Atlantic Forest, is the only species of the monophyletic Rhodniini tribe that occurs in ES^6^. Clarifying the geographic distribution of *R. domesticus* helps to understand the epidemiological and sylvatic cycles of Chagas disease as well as the evolutionary and endemism patterns in the Rhodniini group.
